# Kikuchi-Fujimoto Disease: A Rare Cause of Fever and Generalized Lymphadenopathy

**DOI:** 10.7759/cureus.105444

**Published:** 2026-03-18

**Authors:** Eihab A Subahi, Mahmoud Osman, Elmunzer A Ibrahim, Ammar Musa, Abdalla Fadul, Bara Wazwaz, Ijaz Kamal

**Affiliations:** 1 Internal Medicine, University of Toledo, Toledo, USA; 2 Internal Medicine, Hamad Medical Corporation, Doha, QAT; 3 Geriatrics and Long-Term Care/Internal Medicine, Hamad General Hospital, Doha, QAT; 4 Pathology, Hamad Medical Corporation, Doha, QAT

**Keywords:** differential for fever of unknown origin, kikuchi-fujimoto's disease (kfd), necrotizing histiocytic lymphadenitis, rare cause of diffuse lymphadenopathy, unexplained fever and lymphadenopathy

## Abstract

Kikuchi-Fujimoto disease (KFD), recognized as histiocytic necrotizing lymphadenitis, is an uncommon, non-threatening, and self-resolving condition mainly seen in young adults. It frequently appears with cervical lymphadenopathy and fever, often resembling infectious, autoimmune, or malignant disorders like lymphoma. KFD poses a diagnostic challenge because of its nonspecific clinical and radiological characteristics, potentially resulting in unwarranted tests and intense treatments. We report the case of a 25-year-old male patient who presented with a 12-day history of ongoing high-grade fever, along with vomiting and a dry cough. Preliminary laboratory tests indicated leukopenia and increased inflammatory markers, whereas imaging examinations ruled out acute abdominal issues. Despite broad-spectrum antibiotics, daily fever spikes continued, leading to a comprehensive investigation for pyrexia of unknown origin. Tests for infectious origins, such as tuberculosis, brucellosis, malaria, and viral factors, were negative. Computed tomography of the chest showed mediastinal and hilar lymphadenopathy, while neck ultrasound identified several bilateral cervical lymph nodes, with some exhibiting loss of fatty hilum. Excisional biopsy of cervical lymph nodes revealed geographic necrosis with karyorrhectic debris, paracortical enlargement, and lack of neutrophilic infiltration, indicative of necrotizing histiocytic lymphadenitis. Flow cytometry and immunohistochemistry ruled out cancer, and a diagnosis of KFD was made. The patient showed improvement with supportive care and was released with follow-up appointments in outpatient rheumatology. The follow-up autoimmune evaluation yielded negative results. Thus, KFD should be taken into account in young individuals showing unexplained fever and lymphadenopathy once infectious and malignant reasons have been ruled out. The definitive diagnosis depends on excisional biopsy of lymph nodes and histopathological analysis. Identifying this entity is essential to prevent misdiagnosis, unnecessary treatment, and patient distress. Due to its link to systemic lupus erythematosus, prolonged monitoring is advised. KFD is a significant differential diagnosis for pyrexia of unknown origin accompanied by lymphadenopathy. Timely histopathological verification guarantees suitable treatment and avoids unnecessary procedures

## Introduction

Kikuchi-Fujimoto disease (KFD), also known as histiocytic necrotizing lymphadenitis, is a rare, benign, and self-limiting condition primarily affecting young adults, especially women, with a higher prevalence in Asian populations [1,2]. First described in 1972 by Kikuchi [3] and Fujimoto et al. [4], the disease predominantly presents as cervical lymphadenopathy, accompanied by systemic symptoms such as fever, fatigue, and night sweats [5,6]. Histologically, KFD is characterized by necrotizing lymphadenitis with abundant histiocytes and plasmacytoid dendritic cells, without neutrophilic infiltration. This distinct pathology differentiates it from mimickers such as lymphoma, systemic lupus erythematosus (SLE), and infectious mononucleosis [1,5].

The exact etiology of KFD remains elusive, though hypotheses suggest it could be triggered by viral infections or an autoimmune response [2,6]. Studies have investigated potential associations with Epstein-Barr virus, human herpesviruses, and parvovirus B19, but no definitive causative agent has been identified [1,5]. There is also a notable link between KFD and autoimmune conditions, particularly SLE, necessitating long-term monitoring for the latter in diagnosed patients [2,6].

Clinically, KFD can mimic serious conditions such as lymphoma, often leading to misdiagnoses and unnecessary aggressive treatments. Diagnosis is confirmed through excisional lymph node biopsy, revealing histopathological findings specific to KFD [5,6]. While treatment is generally supportive, including analgesics and non-steroidal anti-inflammatory drugs, corticosteroids may be required in severe cases or those associated with SLE [1,6]. This case report highlights the need for heightened awareness of KFD to facilitate prompt and accurate diagnosis, avoid undue interventions, and ensure optimal patient outcomes.

## Case presentation

A 25-year-old African-origin man presented to the emergency department with a 12-day history of fever associated with vomiting. The patient had been in his usual state of health when he developed a subjective fever associated with postprandial substernal chest pain and vomiting. Vomit contained food particles without blood or mucus. The patient also reported a dry cough that began shortly after the onset of his symptoms. He had visited his primary care physician, who started him on a three-day course of antibiotics, the name of which he did not recall, without improvement. On his follow-up visit, he was found to have elevated serum lipase and amylase levels, which prompted his referral to the emergency department.

The patient was known to have hypothyroidism, but discontinued his medications six months prior. He denied any alcohol intake or smoking. He had not travelled within the past two years. He mentioned that he had drunk unpasteurized camel milk previously, with the last time being three days before that presentation to the emergency department.

On admission, the patient was febrile with a temperature of 38 °C. He did not appear to be in acute distress. Blood pressure was 112/80 mmHg, and his heart rate fluctuated around the 80s with episodes of tachycardia coinciding with his fever spikes. His abdomen was soft and lax with mild epigastric tenderness. Cardiopulmonary examination was unremarkable. Examination of the lymph nodes was not performed on admission. Laboratory investigations showed a white cell count of 2.6×10³/µL, hemoglobin 14.7 gm/dL, amylase 172 U/L, lipase 113 U/L, and C-reactive protein (CRP) 12.6 mg/L (Table 1).

**Table 1:** Initial blood test result during admission

Investigation	Patient Value	Reference Range
Hemoglobin	14.7	13-17.0 gm/dL
White blood cell count	2.6	4.0-10.0 xl0^3^/uL
Absolute neutrophil count	1.2	2-7 xl0^3^/uL
Platelets	187	150-410 xl0^3^/uL
Urea	1.6	2.5-7.8 mmol/L
Creatinine	65	62-106 umol/L
C-reactive protein	12.6	0-5 mg/L
Lipase	113	13-60 U/L
Amylase	172	13-53 U/L
Anti-nuclear antibody	Negative	Negative

**Figure 1:**
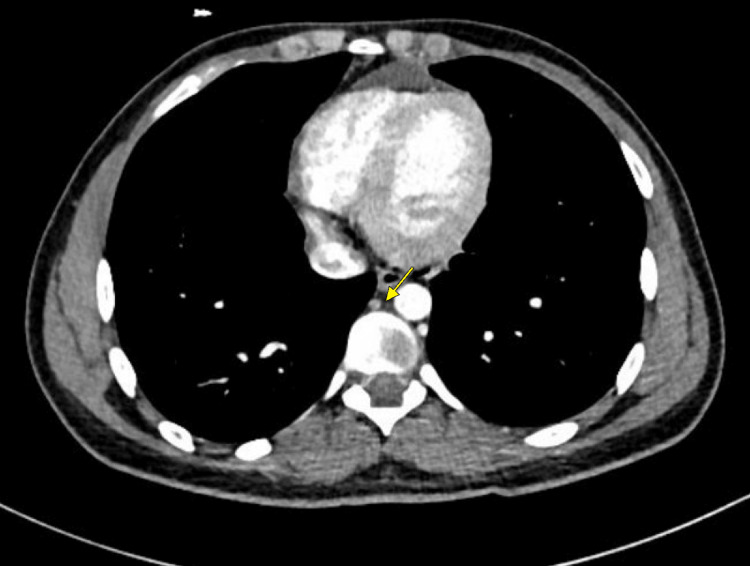
A few, small, subcentimeter prevascular lymph nodes (yellow arrow) are noted

**Figure 2:**
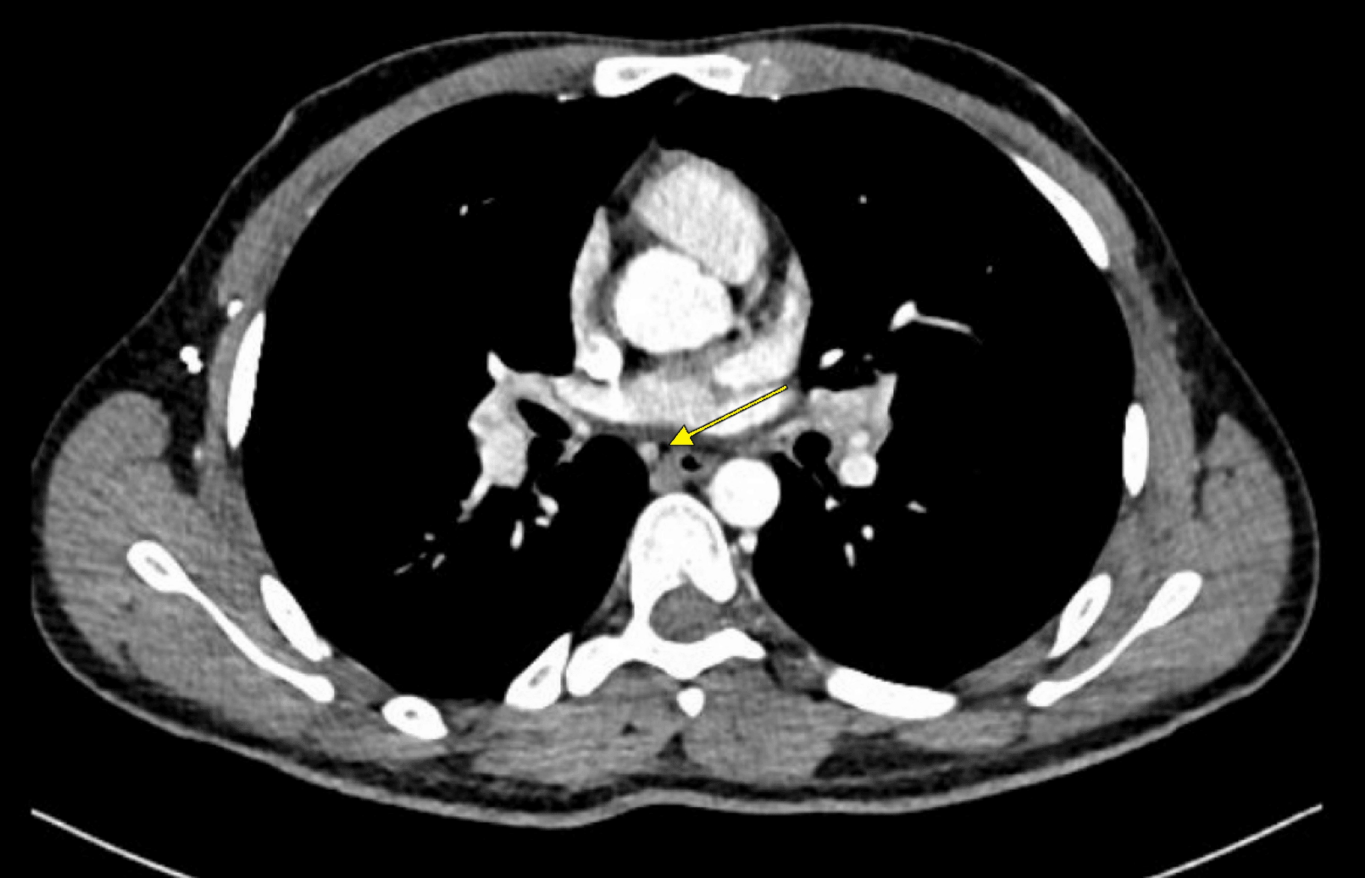
Small bilateral hilar lymph nodes are noted, measuring approximately 11 x 11 mm (yellow arrow).

The patient was euthyroid. Computed tomography (CT) of the abdomen was done to rule out causes of a surgical abdomen, including pancreatitis or appendicitis. It was unremarkable. The patient continued to spike daily high-grade fever around 39 °C despite a five-day course of ceftriaxone, which prompted his investigation for a fever of unknown origin. Workup for tuberculosis, including QuantiFERON (QIAGEN N.V., Hilden, Germany) as well as two sets of acid-fast bacilli smear and tuberculosis polymerase chain reaction (PCR), urine microscopy, extensive respiratory viral panel, brucella and malaria serology, and anti-nuclear antibodies, was negative. CT thorax revealed multiple small hilar and mediastinal lymph nodes measuring around 11 x 11 mm with a few small subcentimeter prevascular and axillary lymph nodes (Figures 1, 2). 

**Figure 3:**
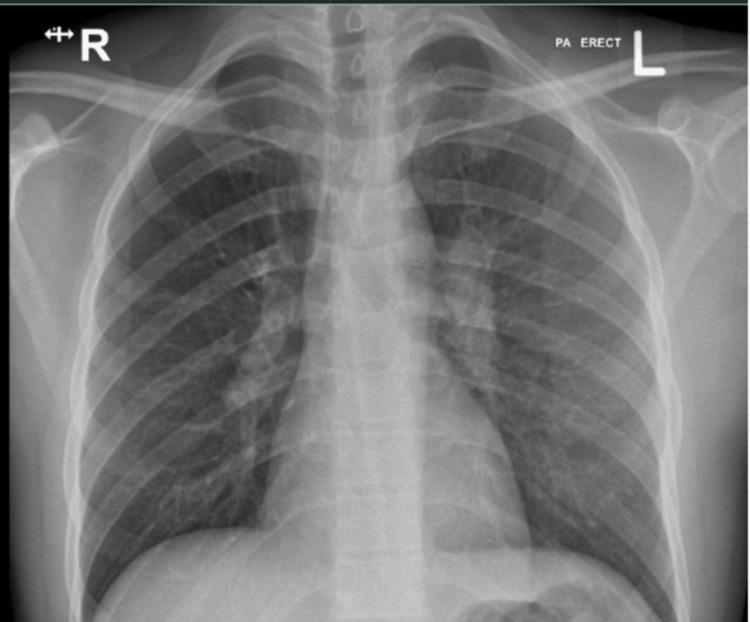
Chest X-ray showing bilateral hilar fullness

**Figure 4:**
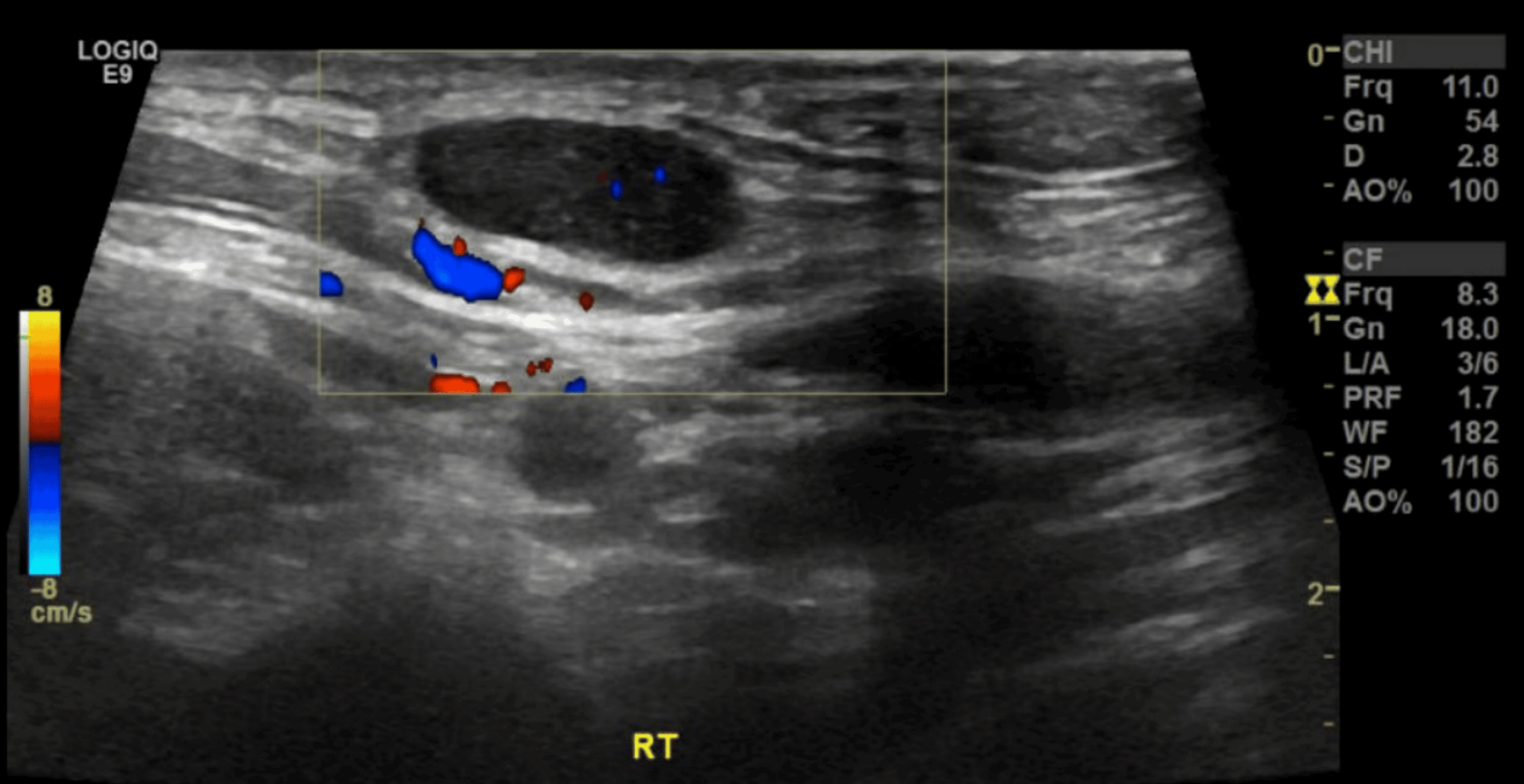
Right upper anterior neck cervical lymph node measuring 12 x 5 mm (“Area of concern”)

**Figure 5:**
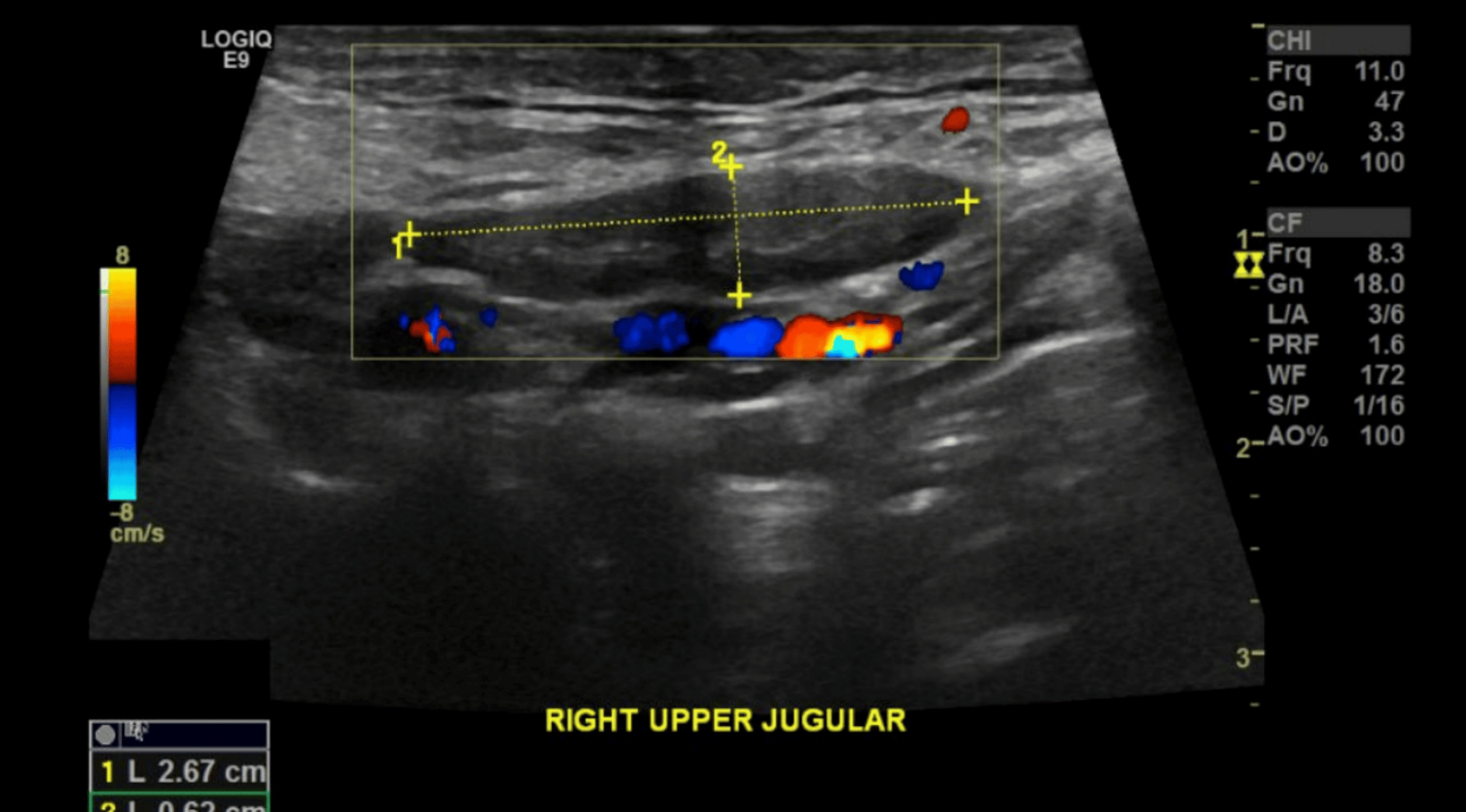
Right upper jugular lymph node measuring 26.7 mm x 62 mm

Chest X-ray on admission was reported to have slightly increased bronchovascular markings and Hilar fullness, which was more pronounced on the left side (Figure 3). Ultrasonography of the neck showed multiple bilateral cervical lymph nodes with a few lymph nodes losing their fatty hilum. The largest of the lymph nodes was 26.7 x 6 mm in size, located in the right upper jugular region. Of note, one lymph node measuring 12.4 x 5 mm was labelled as an area of concern by the radiologist due to its location in the right upper anterior neck (Figures 4, 5). 

**Figure 6:**
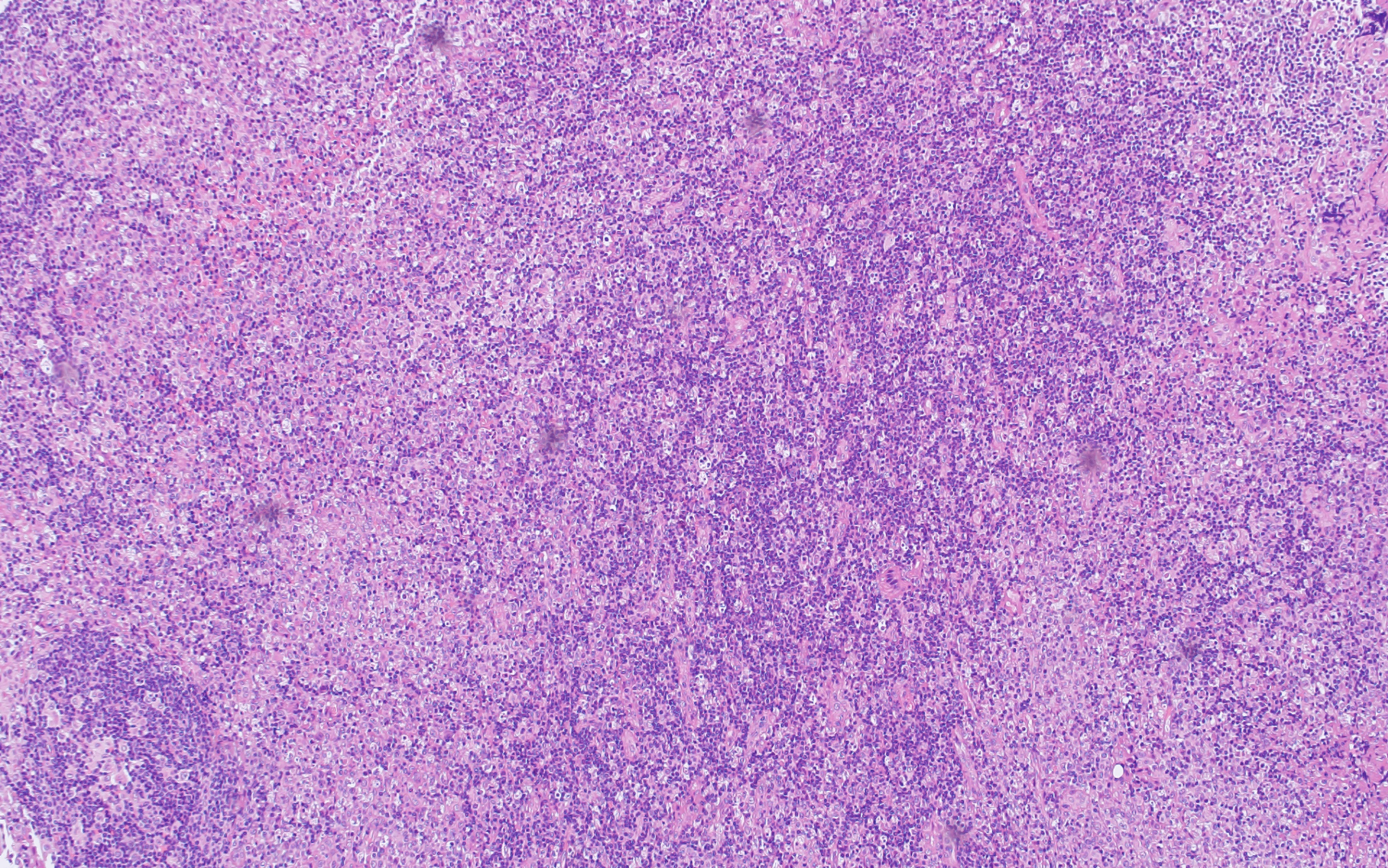
Lymph node excision biopsy with geographic areas of necrosis in a background of reactive secondary follicles, mild paracortical expansion, patent sinuses and some capsular and stromal fibrosis. Neutrophils and granulomata are not a feature.

**Figure 7:**
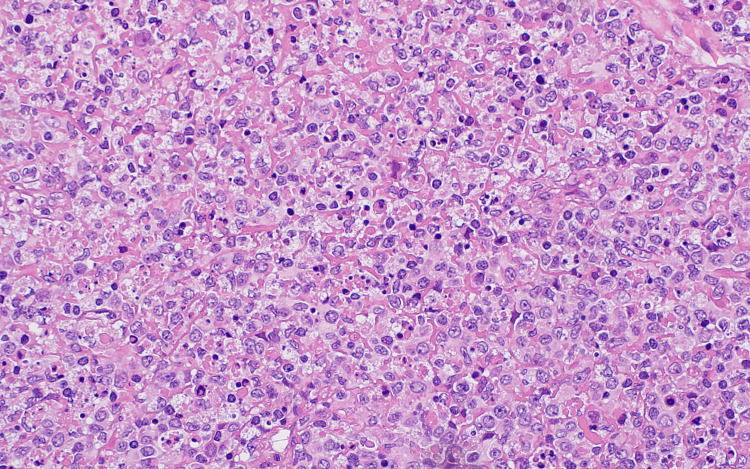
Lymph node excision biopsy showing a higher magnication of areas of necrosis in a background of reactive secondary follicles, mild paracortical expansion, patent sinuses and some capsular and stromal fibrosis. Neutrophils and granulomata are not a feature.

An excisional biopsy was performed on two left cervical lymph nodes, which were sent for flow cytometry and histopathological analysis. Light microscopy showed geographic areas of necrosis in a background of secondary follicles, mild paracortical expansion, patent sinuses, and some capsular and stromal fibrosis with no evidence of malignancy (Figures 6, 7). 

Flow cytometry showed approximately 70% T-Cells with expression of CD3, CD5, and CD7 (majority) with a CD4:CD8 ratio of approximately 2.3:1. Approximately 23% of B-cells expressed CD19 and CD20 with a kappa: lambda ratio at approximately 1.6:1. There was no definitive immunophenotypic evidence of a monotypic B-cell population. Immunohistochemistry staining of the excised lymph nodes showed a reactive phenotype in the germinal centres in the secondary lymphoid follicles (BCL2- with expression of BCL6 and CD10) and IgD expression in primary follicles. There was no aberrant CD5 expression in the mantle zones or outside. Paracortical T-cells expressed CD3/CD5 without abnormal forms. CD21 and CD23 highlight FDC meshworks that are largely preserved with only focal disruption and no expression of these markers in the lymphocyte. CD68 highlights the granulomata. S100 highlights histiocytes. Kappa and lambda do not show any restriction of light chains. The diagnosis of necrotising histiocytic lymphadenitis (KFD) was ultimately made.

The patient was discharged after his fever subsided, following an uneventful postoperative period. At the one-week follow-up to review the results of his biopsy, further SLE and rheumatoid arthritis workup, including rheumatoid factor, anti-citrullinated protein peptide, anti-neutrophil cytoplasmic antibody (ANCA), C3 and C4, and repeat ANA, were done, which were all negative. The patient was discharged on ibuprofen 400 mg three times a day as needed for fever and metoclopramide and referred to the Rheumatology clinic for further follow-up.

## Discussion

KFD, first described in 1972 by Kikuchi [3] and Fujimoto et al. [4], is a rare, self-limiting condition characterized by histiocytic necrotizing lymphadenitis. Although initially thought to be confined to Japan, KFD has now been reported globally, with a higher prevalence in Asian populations. It predominantly affects young adults aged 20-35 years, with a notable female predominance, and has an estimated female-to-male ratio of 4:1 [1,2].

The disease's rarity and overlap with other conditions, such as lymphoma, tuberculosis, and autoimmune diseases, make it a diagnostic challenge. The exact etiology of KFD remains elusive, though two major hypotheses have been proposed: infectious and autoimmune. The infectious hypothesis is supported by the occasional detection of viral particles in affected lymph nodes, including EBV, human herpesvirus 6 (HHV-6), and parvovirus B19. However, these associations have been inconsistent across studies, suggesting that the disease might not be directly caused by pathogens but rather triggered by a dysregulated immune response [7,8]. The autoimmune hypothesis stems from the frequent association of KFD with SLE. Up to 32% of KFD patients develop SLE features over time, and the histological similarities between KFD and lupus lymphadenitis support this link. Moreover, a subset of patients presents with positive ANA and other serological markers suggestive of an underlying autoimmune process [9,10].

Histologically, KFD is characterized by necrotizing lymphadenitis with karyorrhectic debris and crescentic histiocytes. The absence of neutrophilic infiltration or granulomas distinguishes it from infectious causes such as tuberculosis. The findings of plasmacytoid dendritic cells and S100-positive histiocytes further support its immune-mediated pathogenesis [11]. The hallmark presentation of KFD includes tender cervical lymphadenopathy, often unilateral, accompanied by low-grade fever. These symptoms are frequently mistaken for malignancy, leading to unnecessary diagnostic procedures. Systemic symptoms such as night sweats, fatigue, and weight loss are common, further mimicking conditions like lymphoma [1,12]. In rare cases, extranodal involvement has been documented, affecting organs such as the skin, bone marrow, and central nervous system. Dermatological manifestations, including erythematous plaques and nodules, are seen in up to 40% of patients with extranodal disease [11]. Neurological involvement, although extremely rare, can present as aseptic meningitis or encephalitis, further complicating the diagnostic process [7].

The diagnosis of KFD is challenging due to its nonspecific clinical presentation and the overlap with infectious, autoimmune, and neoplastic conditions. Laboratory findings are usually nonspecific and may include mild leukopenia, anemia, and elevated inflammatory markers such as erythrocyte sedimentation rate (ESR) and CRP [1]. Although imaging studies, such as ultrasound and CT, are often employed, their utility is primarily limited to assessing lymph node size and architecture. Common findings include hypoechoic lymph nodes with preserved hilum on ultrasound and homogeneous lymphadenopathy without necrosis on CT. However, these findings are not diagnostic and are often misinterpreted as reactive or neoplastic [7,11]. The definitive diagnosis of KFD relies on histopathological examination of an excisional lymph node biopsy. This approach allows for the identification of necrotizing lymphadenitis with karyorrhectic debris, crescentic histiocytes, and plasmacytoid dendritic cells. Immunohistochemical staining may reveal S100-positive histiocytes, while special stains and cultures for infectious agents (e.g., acid-fast bacilli and fungal elements) remain negative, excluding conditions such as tuberculosis and fungal infections. Fine-needle aspiration cytology (FNAC) is less effective and often inadequate for confirming the diagnosis [2,11].

While imaging is not diagnostic, its role in ruling out other causes of lymphadenopathy cannot be understated. CT findings in KFD typically include cervical lymphadenopathy with preserved nodal architecture and the absence of necrosis, distinguishing it from lymphoma and infectious etiologies. Positron emission tomography (PET)-CT is occasionally utilized but often shows nonspecific uptake, leading to potential misdiagnosis of malignancy [9]. Thus, imaging should be complemented by histological evaluation to avoid diagnostic pitfalls.

KFD is generally a self-limiting condition, with spontaneous resolution occurring within one to four months in most cases. Supportive therapy is the cornerstone of management. Nonsteroidal anti-inflammatory drugs (NSAIDs) are commonly prescribed to alleviate fever and lymph node tenderness. Corticosteroids are reserved for severe cases, particularly those with extranodal involvement or refractory symptoms [6,12]. Given the association between KFD and autoimmune diseases, particularly SLE, long-term follow-up is recommended. Periodic monitoring for serological markers of autoimmune disease (e.g., ANA and anti-double-stranded DNA) is essential, especially in patients presenting with atypical features or those with a family history of autoimmune disease. autoimmunity [10]. Relapses are rare but have been reported in approximately 3-4% of cases, warranting continued vigilance [11].

## Conclusions

KFD is a rare but important differential diagnosis in cases of unexplained fever and lymphadenopathy. Its rarity and nonspecific presentation often lead to diagnostic delays and unnecessary interventions. A multidisciplinary approach, combining clinical, radiological, and histological findings, is critical for timely and accurate diagnosis. While the prognosis is excellent for most patients, clinicians must remain aware of its potential progression to autoimmune diseases, necessitating long-term follow-up.

## References

[1] Fadul A, Subahi EA, Ali EA (2022). Kikuchi-Fujimoto disease: a rare cause of pyrexia of unknown origin and cervical lymphadenopathy. Cureus.

[2] Deaver D, Naghashpour M, Sokol L (2014). Kikuchi-fujimoto disease in the United States: three case reports and review of the literature [corrected]. Mediterr J Hematol Infect Dis.

[3] Kikuchi M (1972). Lymphadenitis showing focal reticulum cell hyperplasia with nuclear debris and phagocytosis (Article in Japanese). Nippon Ketsueki Gakkai Zasshi.

[4] Fujimoto Y, Kozima Y, Hamaguchi K (1972). Cervical necrotizing lymphadenitis: a new clinicopathological agent (Article in Japanese). Naika.

[5] Singh JM, Shermetaro CB (2019). Kikuchi-Fujimoto disease in Michigan: a rare case report and review of the literature. Clin Med Insights Ear Nose Throat.

[6] Sousa Ade A, Soares JM, Sá Santos MH, Martins MP, Salles JM (2010). Kikuchi-Fujimoto disease: three case reports. Sao Paulo Med J.

[7] Hutchinson CB, Wang E (2010). Kikuchi-Fujimoto disease. Arch Pathol Lab Med.

[8] Sopeña B, Rivera A, Vázquez-Triñanes C (2012). Autoimmune manifestations of Kikuchi disease. Semin Arthritis Rheum.

[9] Kwon SY, Kim TK, Kim YS, Lee KY, Lee NJ, Seol HY (2004). CT findings in Kikuchi disease: analysis of 96 cases. AJNR Am J Neuroradiol.

[10] Goldblatt F, Andrews J, Russell A, Isenberg D (2008). Association of Kikuchi-Fujimoto's disease with SLE. Rheumatology (Oxford).

[11] Ahmed Z, Quadir H, Hakobyan K, Gaddam M, Kannan A, Ojinnaka U, Mostafa JA (2021). Kikuchi-Fujimoto disease: a rare cause of cervical lymphadenopathy. Cureus.

[12] Moyer A, Hanafi MZ, Scordino T, Bronze M (2019). Kikuchi-Fujimoto disease: an atypical presentation of a rare disease. Cureus.

